# Digital cultural heritage standards: from silo to semantic web

**DOI:** 10.1007/s00146-021-01371-1

**Published:** 2022-01-09

**Authors:** Brenda O’Neill, Larry Stapleton

**Affiliations:** Department of Computing and Mathematics, INSYTE Centre, Waterford Institute of Technology, Waterford, Ireland

**Keywords:** METS metadata, Semantic web, Open-linked data, Metadata aggregators, Silo, MARC

## Abstract

This paper is a survey of standards being used in the domain of digital cultural heritage with focus on the Metadata Encoding and Transmission Standard (METS) created by the Library of Congress in the United States of America. The process of digitization of cultural heritage requires silo breaking in a number of areas—one area is that of academic disciplines to enable the performance of rich interdisciplinary work. This lays the foundation for the emancipation of the second form of silo which are the silos of knowledge, both traditional and born digital, held in individual institutions, such as galleries, libraries, archives and museums. Disciplinary silo breaking is the key to unlocking these institutional knowledge silos. Interdisciplinary teams, such as developers and librarians, work together to make the data accessible as open data on the “semantic web”. Description logic is the area of mathematics which underpins many ontology building applications today. Creating these ontologies requires a human–machine symbiosis. Currently in the cultural heritage domain, the institutions’ role is that of provider of this  open data to the national aggregator which in turn can make the data available to the trans-European aggregator known as Europeana. Current ingests to the aggregators are in the form of machine readable cataloguing metadata which is limited in the richness it provides to disparate object descriptions. METS can provide this richness.

## Introduction

This paper looks at the standards associated with the digital cultural heritage area at present and also the potential of an emergent standard called the Metadata Encoding and Transmission Standard (METS) to provide an interoperable solution for the semantic web (LOC 2021a). In the cultural heritage domain, the boundaries of institutions, such as galleries, libraries, archives and museums (GLAM), are blurring. All find themselves at a digital precipice which can be traversed by joining forces with what have been until relatively recently, disparate disciplines, to create a viable process for digitisation on the semantic web and the ability to share information openly. Interdisciplinary engagement is crucial to move forward in this area.

This paper is important to those working in GLAM institutions who want to break out of the disciplinary ‘silos’ and data ‘silos’ and reap the benefits of the semantic web. This paper is important to those who want to open up closed archives or archives which are difficult to access. It is also of importance to anyone working or researching in the area of metadata standards and interoperability between systems.

Tim Berner’s Lee vision of the “Semantic web” is getting closer as open data is published on the web and linked open data sets are created. Shadbolt et al. (2006) state “… we see the use of ontologies in the e-science community presaging ultimate success for the semantic web—just as the use of HTTP within the CERN particle physics community led to the revolutionary success of the original Web”.

The COVID-19 statistics that we hear every evening on the news are drawn from open data sets. Governments are now increasingly publishing open data sets and, indeed, Ireland is to the forefront in this regard. It has ranked amongst the “trend-setters” in the Open Data Maturity Report for the past three years. This report serves as a benchmark to gain insights into the developments achieved in the field of open data in Europe (European Data Portal 2020).

From a contextual point of view, it is important to understand that there is a fundamental shift now from traditional closed world modelling of databases to modelling open world systems for the semantic web. These open world systems are created through the use of knowledge systems engineering based on descriptive logic using the web ontology language OWL (OWL 2021), universal resource indicators (URIs) and the creation and publication of data sets on the semantic web which can then be linked to enable contextually rich descriptions of digital cultural heritage objects.

The world has changed. Organisations are now interconnected, inter-dependable, diverse and adaptable. Managers continuously inhabit that area on the continuum from order to chaos at ‘the edge of chaos’. A complex organisations ability to adapt and learn leads to the emergence of complex adaptive systems (CAS). These systems evolve in such an organic manner that they are even described as ‘eco systems’. These systems value highly the creativity, innovation and tacit knowledge of the human. In addition to these organisational changes and with complexity both on a micro- and macro-level—it is no wonder that GLAM institutions find themselves in a position of genuinely wanting to move towards open data but really not quite sure what their new role is or how this metamorphosis is to take place.

Change is occurring on multiple fronts as can be seen through organisational changes, the need to break disciplinary silos and data silos, and the pressing need to liberate knowledge stored in closed or difficult to access archives.

Outline of paper:

The paper starts with Sect. 1 which provides both contextual background and an initial overview of the paper itself.

Section 2 followed by its subsections highlights the current situation.

Section 2.1 illustrates the limitations of both the prolifically used MARC metadata standard coupled with closed world databases. It illustrates the importance of breaking out of data silos by adopting an appropriate common standard, adopting an open world mindset and moving towards cataloguing for the semantic web.

Section 2.2 shows that in the digital cultural heritage sector there is a need to break out of the traditional disciplinary silos and highlights the need for adopting a methodology suited to this kind of work.

Section 2.3 describes how open data sets are currently being created using the MARC metadata standard. The data is supplied by the libraries to the national aggregator who in turn supplies the datasets to the European aggregator.

Section 2.4 describes the benefits of Big Data and data analytics to the use of data-driven methods in libraries.

Section 2.5 shines a light on the mathematics behind ontology building—description logics, and introduces the open source ontology building software called Protégé. It acknowledges the work of John Mc Carthy in the area of description logics, fuzzy logic and cognitive maps.

Section 3 with an eye to the future leads the reader to the METS metadata standard and its potential to provide an interoperable solution to the semantic web.

Section 3.1 describes the criteria this common standard needs to meet to be of value in the future.

Section 3.2 brings the reader to PREMIS and the digital cultural heritage lifecycle (DCC) model and then introduces a theoretical framework emerging from the authors work in the I-CRL which puts the human squarely at the centre of the activity in contrast to the DCC model.

Section 4 guides the reader towards a methodology and framework for interdisciplinary work.

Section 4.1 introduces the human-centred systems (HCS) approach of evolving the technology with the human to produce a human–machine symbiosis.

Section 4.2 introduces the concept of holons, explains how the idea of holons first emerged and how holons can be used to conceptualise complex systems. It links the Soft Systems Methodology and the use of the CATWOE and BATWOV techniques for identifying important holons in a system.

Section 4.3 introduces the reader to the power shift inherent in action research when a multidisciplinary team work together to co-evolve the technology.

Section 5 introduces a methodology to suit interdisciplinary team work. It introduces the concepts of hard systems methodology (HSM) and soft systems methodology (SSM) and then introduces participation action research (PAR). It shines a light on PAR and illustrates its use in indigenous communities, in particular in northern Canada. It describes safeguards that were put in place for the safety of the indigenous communities in involved in research and shows how a community metadata framework surfaced. In relation to safeguards for indigenous communities, it looks at cultural protocols and the use of traditional labels (TK). Having highlighted the sensitivity required in PAR, it introduces the ENRICHER methodology which has emerged from the work of the Insyte-Cooley Research Lab (I-CRL).

Section 5.1 explains how the ENRICHER methodology is operationalised. It then re-introduces the concept of the Metadata and Encoding Standard (METS) as an interoperable solution for the semantic web. It introduces the WikiLibrary Manifesto and the use of FAIR data principles and describes relevant research of the Library of Congress (LC) Online Computer Library (OCLC) researchers.

Section 6 the conclusion reviews what has gone before and ends by emphasising the importance of interoperability and interdisciplinary work leading to a human machine symbiosis within the digital cultural heritage area for the semantic web.

## The current situation

### The need to breakout of the data silos

The standard used by most libraries now is the Machine Readable Cataloguing (MARC) metadata standard (LOC 2021b). The free software tool commonly used by libraries for editing is MARCEDIT (MarcEdit 1999).

The drawback with MARC is that, even though it has been widely adopted in the past for describing books, it lacks richness for describing disparate objects. This richness is required by GLAM institutions now as not only a cataloguing of books is required but also a cataloguing of disparate artefacts in collections, e.g. video tapes, photographs, artefacts in special collections like 35 mm slides, sculptures, tapestries to name but a few.

This coupled with the tsunami of born digital artefacts in the form of electronic correspondence alone that are increasing day on day presents a major challenge and the reliance on digital during COVID-19 has amplified this.

Library cataloguing is changing—the issue to be mindful of now is that there are huge opportunities and benefits to be gained by curating and cataloguing for the semantic web. Operating within a closed world data silo or indeed a network of connected silos was “of its time” but is not the way of the future. Institutions can look ahead now to opening up their data and to do this requires the adoption of common standards.

### Interdisciplinary teams: the need to break out of the disciplinary silos

Interdisciplinary teams are required to work in the cultural heritage area—teams consisting of the knowledge experts (librarians/curators) and technology experts (developers). The tacit knowledge of the team members needs to be valorised. Tacit knowledge is often known as “silent” knowledge or as Polanyi (2009) describes it “that which we know but cannot tell”. This kind of knowledge is sticky, embedded and situated.

Ciborra (1999) brought forth the idea of welcoming in the technology as an invited guest. The co-evolution of the technology is at the heart of all human-centred systems. This involves a double learning loop where team members from various disciplines listen and learn from each other. The building of relationships is hugely important as is a reflective type of practise where actions taken are reflected upon and learned from in a continual learning loop. A suitable methodology which enables this co-evolution of both the process and technologies is required.

### MARC and cultural heritage aggregators: moving towards open data sets

There are a number of cultural heritage information aggregation systems currently e.g. the European Data System (EDM), Open Archives Initiative Object Exchange and Reuse (OAI-Ore 2021), International Council of Museums Conceptual Reference Model (CIDOC-CRM 2021) and an extension of that called FRBRoo (FRBRoo 2021).

A relational ontology for the digital cultural heritage domain was created in the 1990’s by CIDOC-CRM but was deemed to be far too complex for anyone to use. Object-oriented ontologies are used in the CIDOC-CRM aggregator which has a small core of 56 classes. It has become an ISO standard. In any digital repository of artefacts, xml with its extensibility can be used as the common format for potentially all digital descriptions of items being stored (Stapleton et al. 2019).

Doerr (2011) discusses METS, OAI-Ore and CIDOC CRM. He also discusses METS and how mappings can be made between METS and CIDOC. Ahmad and Sharma (2020) speak of the wealth of ancient manuscripts in India that they are just beginning the daunting task of digitising. They also refer to cross walks which is the mapping from one standard to another.

Wijesundara and Sugimoto (2018) state that "There are many digital archives in cultural domains, but there is no well-established metadata model which covers both tangible and intangible cultural heritage." In conjunction with this, there is no well-established metadata model for building digital archives from the aggregation of existing cultural heritage information. They present a model called Cultural Heritage in Digital Environment (CHDE) for South and Southeast Asia.

The Open Archives Initiative Protocol for Metadata Harvesting (OAI-PMH) (OAI-PMH 2021) is a model that has been implemented by many aggregators e.g. Europeana for Europe, Digital Public Library of America (DPLA) in the U.S., DigitalNZ in Newzealand, Trove in Australia and Digital Library of India.

Freire et al. (2018) state “implementation of technological infrastructures for data aggregation have high costs and are particularly demanding on the data providing institutions”. They shine a light on the need to reduce both costs and effort to bring more data providing participants to these networks for sustainability of the network.

Concordia et al. (2010) state that Europeana (The European Cultural Heritage aggregator) is not just another digital portal or library but an application programme interface from which many other uses of the data can be derived. They speak of a need for a change of mindset “instead of trying to sustain the digital information silos of the past, cultural heritage communities are ready for an information paradigm of linked data and thus for sharing as much semantic context as possible”.

The Digital Repository of Ireland (DRI) is the Irish national cultural heritage aggregator. Pierantoni et al. (2015) argue that the DRI has many of the characteristics of a science gateway but that social science stands to benefit in the way that traditionally more numerical sciences did. They differentiate between science gateways and the DRI stating “In many science gateways metadata is generated from the automated analysis of data sets, whilst in the DRI, much of the metadata are manually created by specialised users.”

Currently, the DRI ingests the data in MARC format but have expressed an interest in looking towards METS metadata into the future. In Waterford Institute of Technology, the Luke Wadding librarians are members of the DRI and are in the process of preparing the MARC data for submission to the DRI at the moment.

Once the data have been submitted in the required MARC format, the DRI then makes it available to Europeana.

After the various countries in the EU make the data sets available to their national aggregator, then an aggregation of information from these datasets can be made by the European aggregator creating a holistic and rich digital object.

A tool for the preparation of metadata for the Irish National aggregator the Digital Repository of Ireland (DRI) would be of significant value to the librarians.

When preparing data for the DRI, a template is downloadable from their website (in the form of an xsl spreadsheet) which describes the kind of data to be stored in the fields which are mandatory and the fields which are optional (DRI 2019).

### Benefits of data-driven methods used in libraries

Just as large organisations are using big data and data analytics, so too must libraries move in this direction as there are many benefits to be gleaned from the analysis of such data. Showers as early as 2012 states “Adopting a data-driven model for the development and deployment of library infrastructure has the potential to transform the way the library interacts with its users and enables the development of new services. Importantly, such a data-centric approach changes the very nature of how libraries conceive and tackle the problems they face, both now and in the future” (Showers 2012).

The use of Big Data applications provides many benefits and Kamupunga and Chunting (2019) state that the literature review in their study “indicates that Big Data applications in libraries results in the creation of new knowledge and libraries that use big data analytics are more productive and efficient than others.” On top of creating new knowledge it can also create new roles for librarians and information professionals. As the research by Garoufallou and Gaitanou (2021) indicates “that Big Data are a huge opportunity for libraries, as it can lead to the creation of new roles for the librarians and the information professionals”.

### Description logics

There are a variety of “logics” which enable machine systems to intelligently process datasets. Description logics are a family of formal knowledge representation languages. Badder et al. (2002) as far back as 2002 stated that “The emphasis in DL research on a formal, logic-based semantics and a thorough investigation of the basic reasoning problems, together with the availability of highly optimised systems for very expressive DLs, makes this family of knowledge representation formalisms an ideal starting point for defining ontology languages for the Semantic Web.”.

The Web Ontology Language (OWL) is based on description logics. An ontology is the logical link between the human and the machine world. OWL (OWL 2021) is the World Wide Web Consortium (W3C) standard recommended for semantic web ontologies and is pivotal to the growth of the semantic web.

OWL2 has evolved from Ontology Interchange Language (OIL) + DARPA Agent Markup Language (DAML), to OWL (Fensel and Keller 2005). OWL2 is an extension and revision of the 2004 version of OWL.

Protégé software (Protégé 2021) is open source free ontology building software of which there are many. It uses DL.

Machines perform incredible reasoning tasks at high speed. The work of John Mc Carthy, mathematician and computer scientist in the field of AI has brought various conceptualisation and logics ranging from Fuzzy Logic and Cognitive Maps to DL and many others.

## Looking to the future: the mets metadata standard—a richer way of describing disparate cultural heritage objects for interoperability

### What are the criteria that this common standard needs to meet?

Stapleton et al. (2019) evaluated metadata standards under a number of criteria e.g. interoperability, extensibility, ability to describe a wide range of items, descriptive metadata, structural metadata and administrative metadata and in a comparison between MARC, MODS and Dublin Core the METS metadata standard was discovered to be far richer in what it could describe. A key aspect of the METs metadata standard is *flexibility*. The standard needs to be able to describe *any* artefact. A survey by the Digital Repository of Ireland (DRI) in 2012 showed that only 6% of institutions were using METS at the time (Digital Repository of Ireland 2012). METS meets the flexibility criteria as it is extensible meaning that a description of an item can be extended beyond current schema that may be available to describe objects. METS is XML compliant (W3C 2021) which means that it complies with an important part of the semantic web stack. This gives METs huge flexibility; however, writing a METS description also requires the writing of a Profile (LOC 2021c) to describe the legal entities in that METs description. The Library of Congress (LOC) in the United States manage the METS standard and the validation process that goes with it (LOC 2021d). Creating a Profile means that when the METs description is validated there is also an account of how that METS description was created for that particular object. Other institutions who need to share a similar object can then look at the Profile hosted on the LOC website and create their METs description to conform to that. There is no one common trans-institutional way of describing an item—each item has its own institutional description (unless the institution adopts one of those posted on the LOC website). This flexibility is what makes METS a potential solution to the semantic web interoperability problem.

Gartner (2008) states that the use of a Profile creates an added task for the METS user and discusses the abolition of this task and the strengthening of the standard but acknowledges and warns that to do this would greatly diminish the flexibility that it offers. There is clearly a trade-off between flexibility and reducing the work involved but any reduction in flexibility would be detrimental to the standard as an interoperable solution to the semantic web.

The use of namespaces is central to the inter-operability of the METS standard. It enables the "plugging" in of a huge variety of schemas e.g. PREMIS, MIX, VideoMD, XrML, TEI and many others. (See Appendix A).

Just as any physical object can be described using the METs standard, so too can other objects such as born digital artefacts like emails. METS can describe both tangible (physical items) and intangible cultural heritage (stories behind the items) as specified by the 1972 EU convention (UNESCO 1972).

### PREMIS and the Digital Curation LifeCycle Model (DCC)

**Fig. 1:**
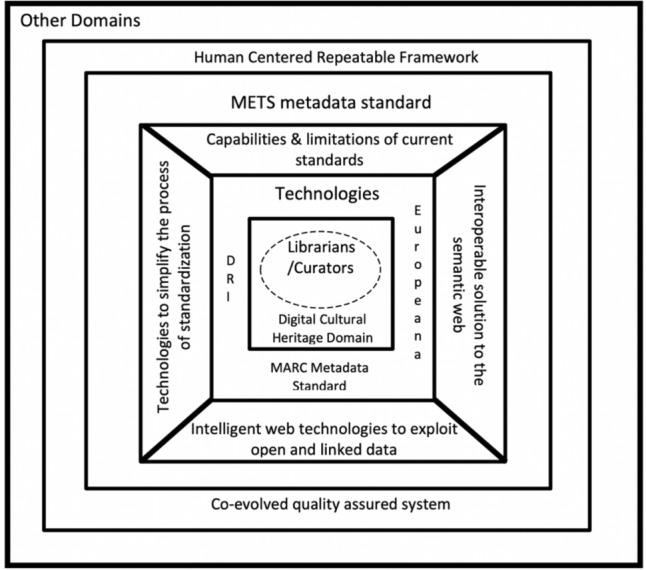
Theoretical human-centered repeatable framework with human at centre

The DCC is very interesting from the point of view of the tasks that are involved in curation and preservation of digital objects. It seems to provide broad brush strokes as to how digital preservation can be accomplished (DCC 2021). It is data-centric and could end up being a “one size fits all’’ solution to digital curation. The only problem with the “one size fits all” solution is that usually it does not fit very well. Each collection is unique and the librarian/curator/archivist/guardian needs to be at the heart of it to bring this uniqueness forward and to tailor the process. Narratives are embodied in the collections and the curators draw these narratives out. A human-centred model which is evolving in the I-CRL is as follows:

Figure 1 places the human at the centre of the process in contrast to the DCC model which places data at the centre of the process. Both the process and the technology are co-evolved.

## Towards a suitable methodology and framework for interdisciplinary work

### A human-centred systems approach and a human–machine symbiosis

Professor Michael Cooley, architect of human-centered systems has written extensively on the human-centred systems approach, particularly in his own discipline of engineering (Cooley 1987, 2018, 2020).

Gill (1997, p. 5) present the ‘Foundational Ideas of Human-centeredness’ amongst which are:

5. Human-centredness is essentially multidisciplinary, crossing academic and cultural boundaries.

8. Tacit knowledge is a cornerstone of human-centred philosophy, rooted in the interdependence between the subjective and the objective, and rejects the notion of their separate existence.

A human-centred systems approach is fundamental to any process that proposes a framework for digitisation of cultural heritage. This approach places the human at the centre of development and the automation/AI augments but does not replace the work of the human. Tacit knowledge is valorised.

The reason for this approach is that the librarians/curators have a wealth of scholarly knowledge accumulated and handed down through generations of care for scholarly artefacts, after all, it is from libraries that the great universities emerged, such as Oxford and Cambridge (Stapleton et al. 2019). Tacit knowledge can be leveraged by computing power. A human–machine symbiosis (Gill 1997) can be formed whereby the human uses the computational power of the machine as the machine ultimately handles this material much better than humans. A symbiosis can be achieved by automating the parts of the work that are suited to this and by liberating the human to guide the direction of work—to use his/her tacit knowledge. Human-centred systems move away from a “Command and Control” hierarchical structure or “transactional” structure which are a legacy of the industrial revolution and Taylorist factory model, to a “No centralised control” system where the knowledge experts and the technologists co-evolve the processes and technologies.

### Holons

A human-centred system is not broken down into subsystems as in a manufacturing system but is viewed in a holistic manner. The various levels of complexity are known as ‘Holons’. A holon can be thought of like a lens which enables the viewer to hone in on the complexity involved in certain parts of the system whilst not being overwhelmed by the complexity of the whole system. The rest of the system is kept in peripheral vision. This holonic view can change just as a lens can change its focus.

Valckenaers et al. (2008) in their discussion of self-organising and self-adaptive systems refer to Simons’ example of the watchmakers (Simon 1990). In brief, there are two individual watchmakers making complex watches of 1000 pieces. When the phone rings to take an order, watchmaker one leaves down the partially assembled watch that he is working on and subsequently has to start again from scratch. The more phone orders received the less watches produced. He eventually loses his shop.

Watchmaker two however has broken down the assembly of the watch according to the various layers of complexity or sub-assemblies if you will. When a phone order comes in, only a sub-assembly component is left down so only a small amount of work needs to be redone. This led to an increase in output of 4000% for watchmaker 2. This productivity rise increases with product complexity and led Simons’ to conclude that the pyramidal structure of holonic systems constitutes a law of the artificial. In a demanding and dynamic environment, a system has to be holistic to survive. It is the humans within the system that make it self-adaptive and self-organising.

Koestler in his description of holons uses the metaphor of Janus who was a two-faced Roman God. He states that the visage looking inwards towards its subordinate parts is that of the whole which is in contrast to the visage looking outwards which is that of a dependent part. To give an analogy: looking inwards a human being is seen as an autonomous, self-contained unit, made up of levels of components/organs inside its body. Looking outwards, it is part of a social community, a county, a state; something bigger than itself, which it contributes to.

Bakos and Dumitrascu (2017) consider how the holonic approach can be used in project risk management of complex adaptive systems to enable the handling of unexpected situations commonly known as “Black Swans”.

Tokody (2018) speaks about digitising the European industry using a holonic systems approach. He describes how with the help of intelligent cyber-physical systems, a holonic (with distributed intelligence) manufacturing technology is developed.

The important thing in soft systems methodologies is to identify the important holons and work on them. CATWOE and BATWOV are examples of methods of identifying these holons (Checkland 1999). Gill (2019) states that “The notion of holon of interest here, is the interconnectedness of relationships between and amongst human systems, between the unit and whole—an interdependent model of the universe, where whole is not sum of parts but inter-connectedness of parts.”

When the holonic focus changes to that of transcribing hand-written correspondence into electronic format, AI could be employed for this but rich information could be lost as AI would not be able to make the connections that the human can from the document to other relevant material that may be in the collection or elsewhere. These insights are an example of the tacit knowledge that Polanyi speaks of. Maybe by, “having eyes” on the digital transcription by quality checking the transcription by the librarian this opportunity for tacit knowledge is preserved, the work of the human is made easier and thus, a human–machine symbiosis is achieved by maintaining the human in the loop.

### A power shift: a move towards action research

The inviting in of the technology leads to a power shift in the relationship away from the developer to the librarian/curator. The librarian/curator, in essence, can request/accept or reject the proposed technology. The multidisciplinary team works together to co-evolve the process and the technology, working in a suitable environment where action research can take place. Observation of tacit knowledge in action is important. A huge influence on Participation Action Research (PAR) has been feminist enquiry into power led by Patricia Mc Guire (Young 2013). O’Neill and Stapleton (2020) suggest that there is a need to move away from Agile and rapid development which has become a “one size fits all” methodology with a disconnect from its’ original ideology.

Agiles’ sequential iterations in itself hinders capitalising on insights which come from the creativity of the human. Creativity can sometimes lead to negating the need for some of the sequential phases initially thought necessary. Human creativity can enable the “dancing across” these phases. The HCS approach emancipates this creativity and innovation and in doing so liberates performance.

## A methodology to suit

There are two types of systems thinking, i.e. hard systems thinking and soft systems thinking. Hard systems thinking is goal-directed and emanated from engineering and the manufacturing of products. Soft Systems thinking emerged from working with problems which are hard to define a problem of a Human Activity System.

According to Checkland a persons’ world view is sometimes known as “Weltanschauung” which means a particular philosophy or view of life; the world view of an individual or group. It is very easy when re-engineering a system to subconsciously apply your own “Weltanschauung” to it (Checkland 1999).

**Fig. 2:**
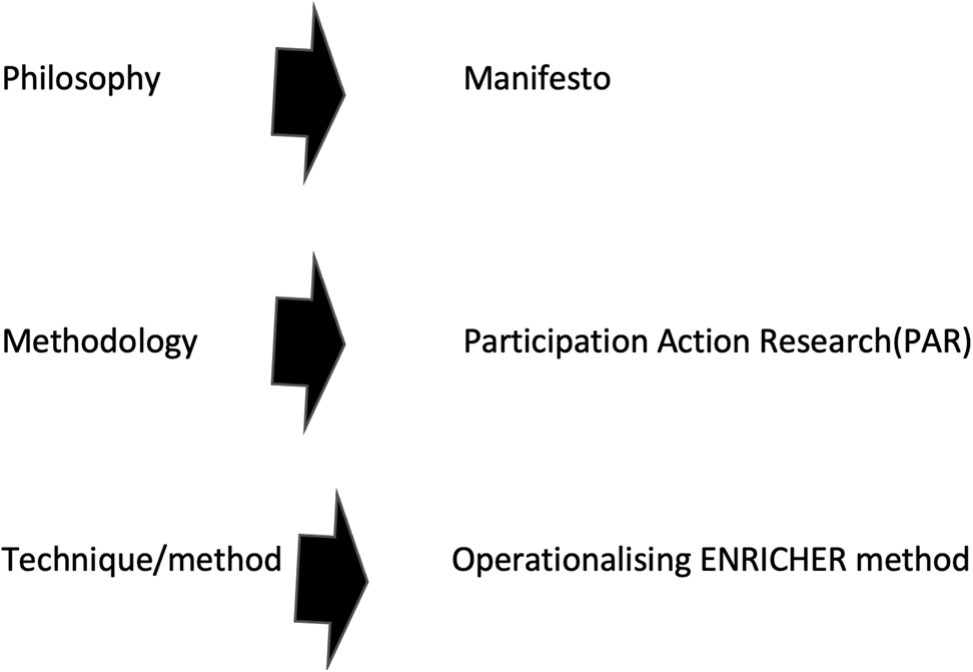
Application of Peter Checklands' view of methodology (Checkland, 1999)

Action research stems from the soft systems methodology. Checkland (1999 p. 162) states “I take a methodology to be intermediate in status between a philosophy, using that word in a general rather than a professional sense, and a technique of method.” His sense of the output of the research is not as a “method but a set of principles of method which in any particular situation have to be reduced to a method uniquely suitable to that particular situation” (Checkland 1999, p. 161). Participation action research (PAR) is a type of ‘meta’ methodology or “*set of principles of method”* which are then moulded to a method uniquely appropriate to each situation. (Checkland 1999) (Fig. 2).

**Fig. 3:**
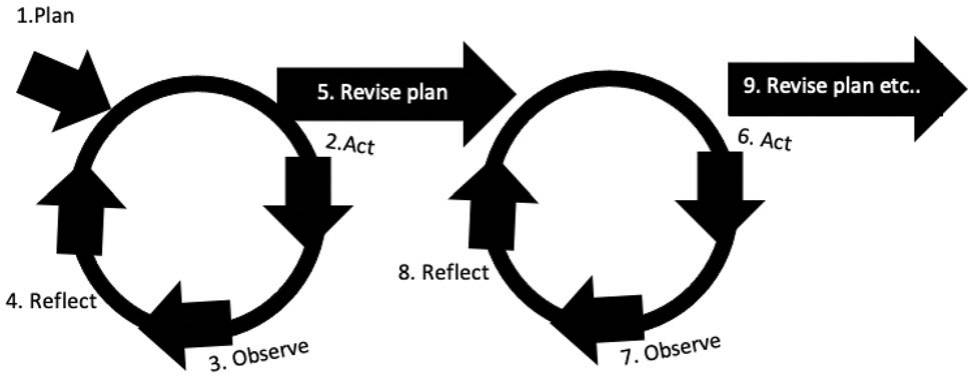
Adapted from the Action Research Spiral (Kemmis and McTaggart, 1982, p.8)

PAR suits multidisciplinary teams who wish to co-evolve the process and technology, as under the umbrella of PAR various methods to suit the particular situation can be taken, e.g. ENRICHER for the Professor Michael Cooley Special Collection (Fig. 3).

Mumford as early as 2001 in relation to Action Research and Socio-Technical Design says “…that they are well worthy of consideration as approaches and tools for the future. They provide opportunities for long-term, in-depth, research which fits well with todays beliefs in a multidisciplinary approach and in organisational democracy” (Mumford 2001). McTaggart (1994) gives a comprehensive account of the theory and practise involved in PAR. He states “Action research remains a diverse and thoroughly justified and preferred mode of educational and social enquiry, continuing to address the concerns of both its practitioners and its critics” (McTaggart 1994). One of the key elements of PAR is engagement. The traditional disconnect between researcher and those being researched is removed. There is no division between both, all are equals—co-researchers. The researcher acts as an “advocate” for the group. Heshusius (1994) states “When one forgets self and becomes embedded in what one wants to understand, there is an affirmative quality of kinship that no longer allows for privileged status”. This is why PAR suits this kind of research.

It is also why PAR plays a very important role in research into indigenous community knowledge. Research like this is highly sensitive. UNESCO (2021) states that “Local and indigenous knowledge refers to the understandings, skills and philosophies developed by societies with long histories of interaction with their natural surroundings.”

Goodman et al. (2018) give a very good account of research experiences of urban indigenous peoples in Vancouver, Canada. A PAR methodology was used with a team made up of indigenous people and academics. The indigenous team members suggested a traditional means of holding “talking circles” where members could pass or could decide to talk on a preselected topic. Each participant as they spoke held up an eagle feather—as was their custom and this was very much appreciated by them.

The research also showed that many indigenous people who participated in research did so because monetary compensation was offered and it provided them with a way to survive in extreme poverty. They called this the “Research Economy”. Many felt they had “been researched to death”. The authors themselves speak of the need for ethical research.

Many of the participants felt a disconnect to the research as Goodman et al. (2018) state “…with the exception of the research presented here, the fact that participants rarely perceived such benefits and instead felt research was for the obscure benefit of “others” emphasises the need for research to be done in a “good way”; this means embracing indigenous approaches to research to ensure individuals and communities are both informed of and touched by research outcomes in ways that foster the empowerment of indigenous peoples in research”.

Because of the over researching, bordering on exploitation of indigenous peoples, using inappropriate Western traditional methods Schnarch (2004) produced an article giving some options for First Nations Communities which encompass ownership, control, access and possession of research, thus giving the power back to the indigenous communities.

Research carried out by Farnel (2020) shows how a metadata framework which was driven by an indigenous community in the North of Canada surfaced. She took a participatory action approach to the research and possessed a deep recognition of peoples differing world views or ways of knowing. Interestingly in it she talks about global metadata standards and how some indigenous communities are really not concerned with sharing their knowledge globally but with creating a valuable resource for their own community (Farnel 2020). She notes “There is no one “Indigenous people”’ there is diversity within diversity” (Farnel 2020, p. 24).

Cultural protocols and traditional labels (TK)

The LOC gives the following definition for traditional knowledge labels (TK) “Traditional Knowledge (TK) Labels are an educational and informational digital marker created by the Local Contexts initiative to address the specific intellectual property needs of Native, First Nations, Aboriginal and Indigenous peoples with regard to the extensive collections of cultural heritage materials currently held within museums, archives, libraries, and private collections.” (LOC 2021e).

Researchers of indigenous communities use these TK labels to identify community access protocols and guidelines. These labels serve as an aid to anyone outside the community in understanding the significance and importance of material even when it is in the public domain (LOC 2021e).

In relation to power, PAR levels the playing field. The Insyte-Cooley Research Lab (I-CRL) is set up as a PAR laboratory for longitudinal studies, with a community of practice made up of the lab members who consist of interdisciplinary team members, such as librarians, academics, researchers, students and industry members, who are all stakeholders in the research and in finding how to work together to advance the work in this area.

A methodology that the I-CRL is using under this PAR umbrella, and which has emerged from the ongoing work in the lab, is called ENRICHER (Stapleton et al. 2020). The ENRICHER method can be seen below:

1. Ethos centric: ethos of development continuously re-visited and reviewed. Important to articulate and re-articulate core values of development.
2. Engagement as an outcome; shift from “why are we doing this” questions to “how are we engaging together on this” question.
3. Reuse machine knowledge: reuse and extending existing knowledge models rather than predefining total schema where possible before implementing.
4. Insights from context: derive technology to fit the context—of tacit knowledge use. means acquiring an understanding of knowledge in action to drive software creation and technology development.
5. Co-evolution: co-evolve the methodology and the technology with all participants. Also, co-evolve and reshape work-technology symbiotic relationship.
6. Hospitality: technology “guest” invited into the work context, otherwise not deployed.
7. Expressiveness: semantics emphasise expressiveness of the machine model rather than processing efficiency and technical capability (which come later).
8. Reverse engineer and extend semantic model: constantly reverse engineer from data and metadata resources and standards as a way of building the knowledge model, extending the model and integrating the resources semantically.

### How is enricher operationalised?

Every fortnight there is a “Show and Tell” meeting where students present the progress that they have made on their research and the members of the lab bring their wealth of experience to bear when they provide feedback.

Regular communication between librarians and developers is very important and a protocol has been agreed upon whereby the author speaks to the head librarian and disseminates the knowledge to the students. The first items being focussed on are the work processes of the librarians and, of course, during COVID-19 adaptations have had to be made as the physical lab is no longer operational so “observation” of the processes has been rendered impossible.

The lab has a space where both researchers and librarians can work together thereby enabling observation or discussion of the work practices normally—this has been adapted so that that librarians can send the information electronically e.g. templates, spreadsheets etc. with a brief description. When conditions permit videos may also be made of the work processes when the librarians are safely back on site.

Other meetings between librarians and author happen as required throughout the process. There is an emphasis on relationship building.

**Fig. 4:**
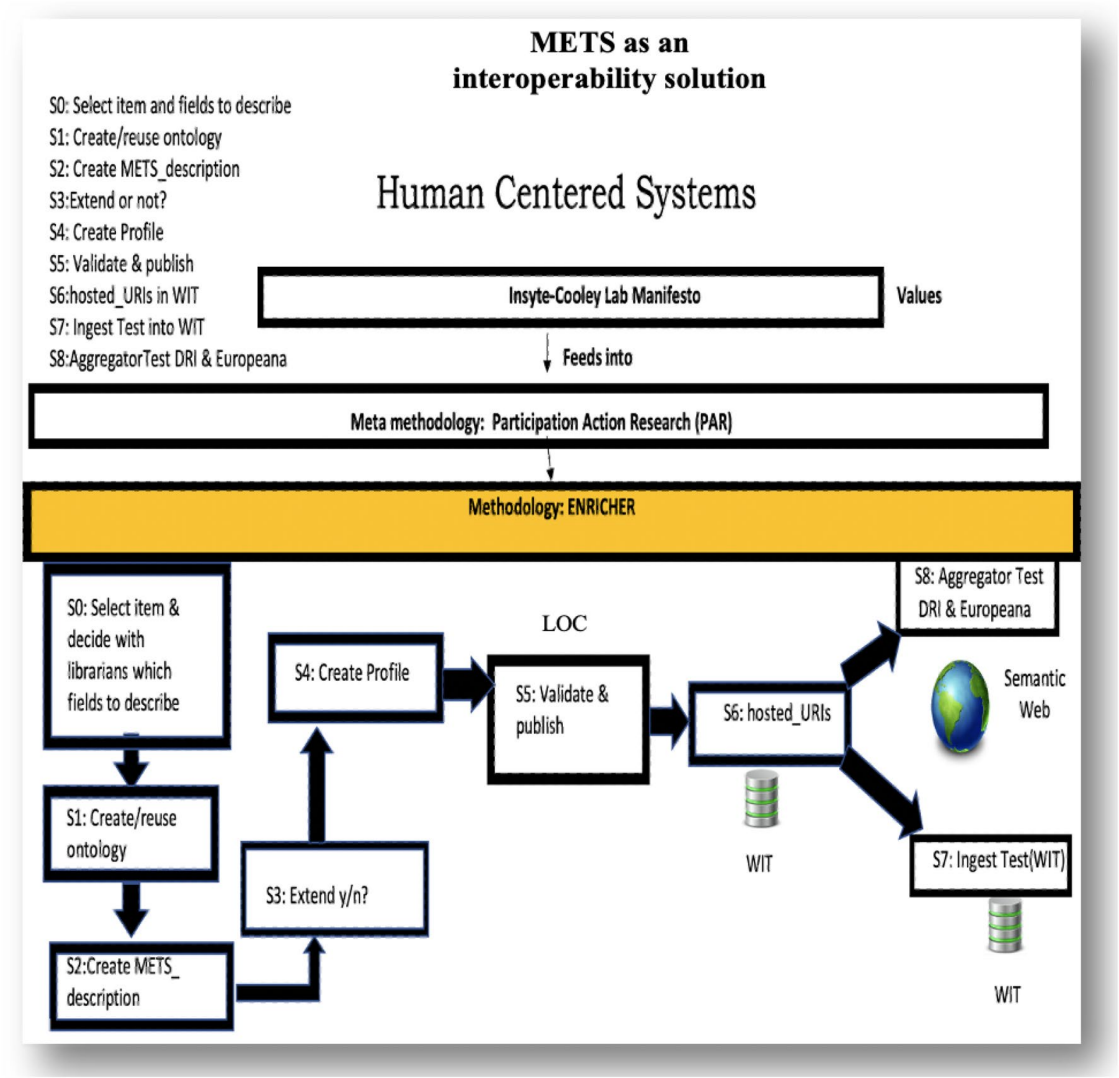
METS as an interoperable solution

A key point of the ENRICHER method and any human-centred method is the co-evolution of the technologies. In Fig. 4 it starts with knowing what fields are required to describe an object. METS enables the use of many schema, e.g. Dublin Core, MARC, MODS. This means that standardised descriptors can be used within METS to describe various aspects of objects. This would enable the setting up/adaption/reuse of an ontology using the Protégé Ontology Language software (Protégé 2021). This requires the co-evolution of a “conceptual model” by the librarians and technologists to gain a common understanding of the domain of discourse and the relationships that exist between concepts. This builds on RDF triples made up of subject, predicate, object each of these resolving to a universal resource indicator (URI) accessible on the semantic web. The web becomes an “open world” database. Once there are URIs created then the next step of the creation of the METS description and profile can be pursued. The METs description has to be validated by the LOC and is then published with the Profile on their website for others to use.

The International Federation of Library Associations and Institutions (IFLA 2021) has endorsed the WikiLibrary Manifesto which aims “at connecting libraries and Wikimedia projects such as Wikibase in an international network of knowledge” (Wikimedia 2020). The WikiLibrary Manifesto uses the FAIR data principles which were first proposed in a paper by Wilkinson et al. (2016) and subsequently revisited again in 2017 (Mons et al. 2017). It is a set of guidelines which focus on **f**indability, **a**ccessibility, **i**nteroperability and **r**eusability of digital assets.

The LC Online Computer Library Center (OCLC) states “Wikidata/Wikibase are viewed as a possible alternative to traditional authority control and have other potential benefits such as embedded multilingual support and bridging the silos describing the institution’s resources” (Smith-Yoshimura 2020).

Bahnemann et al. (2021) describe the Linked Data project. OCLC partnered with institutions that manage their digital collections with the OCLC’s CONTENDdm service. The project used the Wikibase environment and in the course of the project necessary tools were used/built. It demonstrated to the participants the value of linked data as a way of creating and maintaining metadata. One of the five institutes involved in this project stated that “We will use the knowledge gained from this project to rethink our workflows and our descriptive metadata with an eye towards the promise of linked data.” Another stated “The Wikibase offered a glimpse into what a digital collections database that employs linked data might look like and how the cataloguing process might change”. This experience led the participants to the realisation that their workflow practises will undergo change as they re-engineer their cataloguing to suit linked data. In relation to continuing the journey to linked data, the report states “A paradigm shift of this scale will necessarily take time to carry out and calls for long-term strategies and planning.”

## Conclusion

An output from the Archives in the UK/Republic of Ireland and AI (AURA) network and the three workshops it held were that initially people spoke about what they were doing and their current situation; but from the dialogue at the workshops, it is evident that there is the beginning of a shift in understanding of the wider global picture (AURA 2021). There is also a concern about the need for the development of expertise in this area moving forward.

It is important that GLAM institutions move towards open-linked data on the semantic web and break out of the data silos of the past. To do this, disciplinary silos also need to be broken as the humanities and science need to find a way to work together in this challenging domain. The librarians/guardians/curators of artefacts possess vast knowledge about the artefacts under their care. Much of the care that they take with these artefacts is tacit and intuitive. These human characteristics just like creativity and innovation resist datafication. Both Cooley and Gill speak of a human–machine symbiosis—a symbiosis of the objective with the tacit, a symbiotic lens—where the best of the human can be in synergy with the best of the machine. Professor Howard Rosenbrock provided the idea of a ‘Machine with Purpose’ where four alternatives for technology design were put forward. The fourth option was that technology be used for social benefit so that the more beneficial the technology becomes for humanity, the more demand for socially useful technology. Both technology and social benefits expand in a symbiotic relationship (Gill 1997).

It is important to be very clear on what human knowledge is and what machine knowledge is. human knowledge includes tacit knowledge, creativity, innovation, emotional intelligence and causality to name but a few. Machine knowledge consists of computing power but also with the advances in AI the ability to infer from logic. Ontology languages are based on the area of mathematics called description logics. Ontologies enable a human to describe a domain and that domain can be inferred from and utilised by machines. Data-driven methods in the form of big data analytics are being used by libraries to their advantage.

The need for interdisciplinary teams to work together in the area of digitisation of cultural heritage is paramount. There is also a need to co-evolve the process and the technologies together. Action research facilitates this close work.

Initially, research on indigenous people was prolific and because they were living in extreme poverty and were offered a stipend for taking part in surveys was ethically questionable. The situation is improving and research on indigenous people can benefit greatly from a PAR approach particularly in developing community metadata frameworks. The use of Traditional knowledge labels and protocols for access to these communities and their artefacts are very helpful to this field of research and act as safeguards to their cultural assets both tangible and intangible.

The I-CRL is created as a PAR longitudinal study working in the area of cultural heritage. Knowledge systems engineering using descriptive logic enables the human to map a domain (ontology) so that it can be turned into machine-readable language, use URIs to identify it and publish it with open data sets that can be linked to provide contextuality. This is one way of creating a human–machine symbiosis. This feeds into Tim Berner’s Lee idea of a semantic web.

Interoperability is the other big issue of the semantic web and with the use of METS as a common standard this could potentially be overcome.

Aggregators both national and European are at the forefront of publishing open-linked data sets in the digital cultural heritage area. They are currently using MARC as the standard but METS is a potential for interoperability moving forward.

From an academic perspective, particularly in relation to the I-CRL, research in this area is currently being fed back into the college classroom through means of modules for xml, human-centred systems and analytics and knowledge engineering modules. Undergraduate students participate within the lab on their fourth-year projects as do masters students and PhD students; so there is a synergy between the lab and the classroom. These graduates could possibly be recruited by GLAM institutions to provide the expertise required; however, there is also a need to provide training opportunities for upskilling in this area for current staff in GLAM institutions. Jacobs (2016) shows how PAR was utilised within an educational environment and describes the benefits to all involved when using PAR as a methodology.

Finally, it is so important that it be recognised that in cultural heritage, it is all about the stories/narratives and how these are represented for generations to come. It is vitally important that the human is kept at the centre of development and that the technology is wrapped around them. It is a wonder of our age to be able to view digital artefacts on the web, but it is the story behind the artefacts that breathes life into them.

It is possible to tell these stories and to tell them well in the digital age; however, it requires a combination of human and machine and interdisciplinary work to make the information available for everyone on the global database that is the semantic web.

## Data Availability

Not applicable.

## Appendix A

**Table:**

Abbre viation	Name	For	Standards Body	Namespace
MIX	Metadata for images in XML	Still images	Library of Congress, U.S.A. https://www.loc.gov/standards/mix/	
TEI	Text encoding initiative	Text	TEI Consortium https://tei-c.org/	
AudioMD*	Audio metadata technical extension schema	Audio	Library of Congress, U.S.A. https://www.loc.gov/standards/amdvmd/audiovideoMDschemas.html	
VideoMD*	Video metadata extension schema	Video	Library of Congress, U.S.A. https://www.loc.gov/standards/amdvmd/audiovideoMDschemas.html	
PBCore	Public broadcast T.V. services	Video	PBCore.org (U.S.A.)	
ODrL	Open digital rights language	Rights	World Wide Web Consortium (W3C) https://www.w3.org/ns/odrl/2/	http://www.w3.org/ns/odrl/2/
XRmL	Extensible rights mark up language	Rights	xrML.org https://eduworks.com/Documents/Workshops/EdMedia2003/Docs/XrML2_0/xrml2part5.htm	
PREMIS	Preservation metadata maintenance activity	Preservation	Library of Congress, U.S.A.	http://www.loc.gov/premis/v3
